# Introducing the MAVEN Leadership Training Initiative to diversify the scientific workforce

**DOI:** 10.7554/eLife.69063

**Published:** 2021-05-25

**Authors:** Y Claire Wang, Elizabeth Brondolo, Rachel Monane, Michaela Kiernan, Karina W Davidson, The MAVEN Leadership Team, Catherine M Alfano, Betty Diamond, Joan Duer-Hefele, Felicia Hill-Briggs, Jerry Kim, James Peacock, Nancy D Spector, Sunmoo Yoon

**Affiliations:** Feinstein Institutes for Medical Research, Northwell HealthManhassetUnited States; Feinstein Institutes for Medical Research and Donald and Barbara Zucker School of Medicine at Hofstra/Northwell, Northwell HealthHempsteadUnited States; Feinstein Institutes for Medical Research, Northwell HealthManhassetUnited States; Johns Hopkins University School of Medicine, Johns Hopkins UniversityBaltimoreUnited States; Rutgers Business School, Rutgers UniversityNewarkUnited States; Cardiac Electrophysiology Program, White Plains HospitalWhite PlainsUnited States; Executive Leadership in Academic Medicine Program and Drexel University College of MedicinePhiladelphiaUnited States; Columbia University Irving Medical Center, Columbia UniversityNew YorkUnited States; 1 Department of Health Policy and Management,Mailman School of Public Health, Columbia UniversityNew YorkUnited States; 2 College of Liberal Arts and Sciences, St. John’s UniversityJamaicaUnited States; 3 Feinstein Institutes for Medical Research, Northwell HealthManhassetUnited States; 4 Stanford Prevention Research Center, Department of Medicine, Stanford University School of MedicineStanfordUnited States; 5 Donald and Barbara Zucker School of Medicine at Hofstra/Northwell, Northwell HealthHempsteadUnited States

**Keywords:** MAVEN, mentorship, intersectionality, diversity, careers in science, equity diversity and inclusion, None

## Abstract

Addressing gender and racial-ethnic disparities at all career stages is a priority for the research community. In this article, we focus on efforts to encourage mid-career women, particularly women of color, to move into leadership positions in science and science policy. We highlight the need to strengthen leadership skills for the critical period immediately following promotion to associate/tenured professor – when formal career development efforts taper off while institutional demands escalate – and describe a program called MAVEN that has been designed to teach leadership skills to mid-career women scientists, particularly those from underrepresented groups.

## Introduction

Diversifying the research workforce is essential for making the most of public investments in science and medicine, driving innovation, training future scientists, informing public health practices, and achieving health equity (Campbell et al., 2013; Nature, 2018; Institute of Medicine, 2004). Diversifying leadership in the research workforce will also make the outputs of scientific and medical research more relevant to the public at large. However, long-standing gender and racial-ethnic disparities in the research workforce, especially at the leadership level, have resulted from a multitude of cultural, institutional, interpersonal, and individual structural racist and sexist factors – many of which may be unconscious yet ingrained in institutional policies and processes (Kaplan et al., 2018). This paper is the product of a group discussion convened by the MAVEN Leadership Team at the Cold Spring Harbor Laboratory’s Banbury Center in December 2019 to address these issues.

In the United States (US), women receive more than half of undergraduate and postgraduate degrees in the biomedical sciences (Valantine et al., 2016), but men – especially non-Hispanic white men – hold the majority of scientific leadership positions. This paucity of diversity is most striking at the levels of center head, division chief, department chair, and dean, and a recent analysis found that the lowest representation of women and racial-ethnic minorities occurred at the full professor/senior scientist level (Berry et al., 2020; Gold et al., 2020). Women make up only 18% of full professors in the biomedical sciences, and this statistic has remained largely unchanged over the last 20 years (Nikaj et al., 2018; Valantine et al., 2016). Furthermore, when women become department chairs, they make $67,517 less than their male peers even after adjustment for term length, specialty, title, and regional cost of living. They are also more likely to have ‘interim’ in their titles (Mensah et al., 2020).

**Figure 1.**
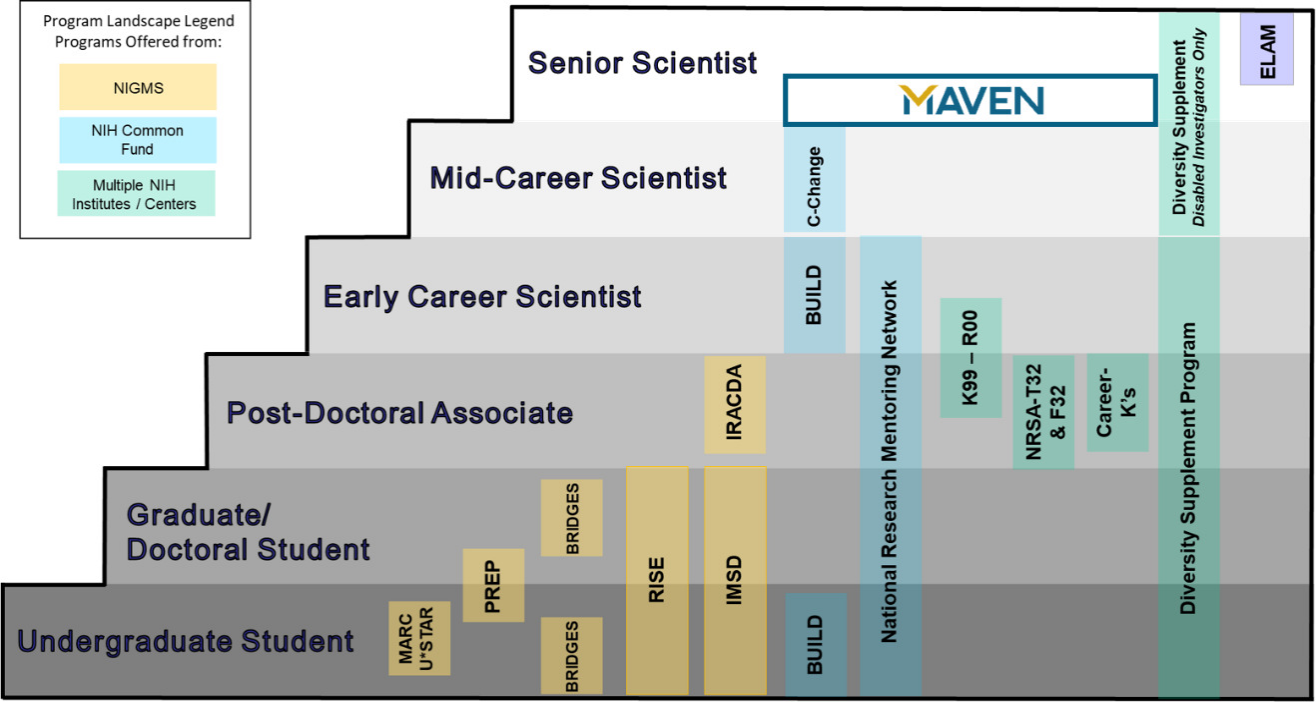
Diversity-related career development activities by career stage. Diversity-related career development activities run by various parts of the National Institutes of Health (NIH) in the US from undergraduate student (bottom row) to senior scientist (top row). The Executive Leadership in Academic Medicine (ELAM) program for senior women scientists who are already in leadership positions is also shown (top right). The MAVEN Initiative is aimed at newly tenured women scientists from underrepresented groups, as very few career development programs are presently targeted at this population.

In the US, funding from the National Institutes of Health (NIH) and other bodies for efforts to diversity the research workforce has contributed to a substantial increase in the number of scientists from underrepresented groups working in biomedical and behavioral science (Valantine et al., 2016). However, funding for efforts to diversify the workforce at senior levels is almost nonexistent (Figure 1). Overall, women represent less than 40% of the NIH-funded investigators at any level and less than 30% of those funded at the Established-Investigator level (Nikaj et al., 2018). A recent analysis of the NIH Director’s High-Risk, High-Reward Research awards suggests that gender inequity remains a concern (Wadman, 2019).

The absence of racial-ethnic minorities receiving NIH grants is particularly striking: approximately 80% of the Principal Investigators (PIs) funded by NIH grants are non-Hispanic White persons. This is not representative of the US demographics, and scientists from African-American, Latinx, and Native American groups are significantly underrepresented among the pool of established PIs. Understanding the causes and developing innovative solutions for these funding gaps is of paramount importance (Stevens et al., 2021; Taffe and Gilpin, 2021).

## Tenured faculty can benefit from training

Studies of career progression have shown that, on the path from postdoctoral fellow to full professor, the highest drop-out rate for women occurs during the transition from associate professor to full professor (Lautenberger and Dandar, 2020). Moreover, career morale and optimism about the future are consistently lowest for tenured scientists and, on average, tenured associate professors rate their career satisfaction worse than both assistant and full professors (Mathews, 2014). These findings point to the critical period immediately following promotion to associate/tenured professor (or a comparable rank) during which training and support are needed, even though everyone who has progressed so far is evidently skilled.

At the Cold Spring Harbor meeting, we reviewed existing training programs for scientists and did not identify any designed to teach leadership skills to those seeking to advance to senior scientist positions. Academic institutions and those who create and/or fund early-career development programs might assume that these newly tenured scientists have received all the training they need – and they may indeed develop the skills they need on their own through serendipity – but many of them require training that is tailored to their needs. Professionals moving into leadership positions need to acquire skills that are quite different than those that helped them grow their research programs.

This lack of support for newly tenured scientists is especially pronounced for women and those from underrepresented groups. The attrition of such faculty at the mid-career stage may also indicate that they have fewer trusted colleagues or role models to provide informal mentoring; this is both a gap and a missed opportunity. Mid-career scientists would also benefit from gaining skills to strengthen their ability to lead others and create a culture of respect (Valantine, 2020). An additional burden faced by women and scientists from underrepresented groups is the likelihood they will be asked to take on duties related to underrepresentation, though such endeavors often count for little in terms of promotion.

Several career development programs have demonstrated improvement in retention and promotion among women in academic medicine in the US. Specifically, a recent review of three such programs – a four-day course for early-career women run by the American Association of Medical Colleges (AAMC), a similar course for mid-career women, and the Hedwig van Ameringen Executive Leadership in Academic Medicine (ELAM) program at the Drexel University College of Medicine – found that they improved 10-year retention in health and medicine careers compared both to peers who did not participate in these programs and to peer groups of men (Chang et al., 2016).

ELAM is a year-long, part-time fellowship that includes person-, organization-, and community-level elements to address barriers to leadership engagement and effectiveness. To date, more than 1,000 ELAM alumnae hold leadership roles (such as department chairs, deans, and other executive ranks) in academic and medical institutions around the world (Jagsi and Spector, 2020). As ELAM focuses on professional development for women who are already in leadership roles, we argue that a comparable mechanism is needed to support women, particularly women of color, who are one stage earlier in their leadership careers.

## The MAVEN Initiative

Building on the premise that scientific leaders are the cornerstone of any academic institution, we envision a diversity-focused leadership program called MAVEN (which is not an acronym) – a word that describes a person with critical knowledge to make a difference. Recently funded by the National Institute of General Medical Sciences (part of the NIH), MAVEN aims to provide mid-career women in science, particularly women of color, with the skills, mentoring, and professional development they need in order to thrive as they advance in their careers. MAVEN is, we believe, unique in the way it addresses the cultural, institutional, and interpersonal barriers to leadership advancement (see http://www.maveninstitute.org for more information).

There are three critical components of MAVEN: the selection process; an intensive skills development program that includes coaching and workshops; and a designed professional network that includes peers and role models. Potential participants will be underrepresented female scientists at US institutions, from all areas of research relevant to medicine or health, who are 10 to 15 years from their final training and are in receipt of substantial federal funding to support their research. To minimize biases in recruitment, we will (i) use the NIH RePORT system to systematically identify women scientists who meet the eligibility criteria, (ii) randomly select and invite stratified cohorts of potential participants from the eligible pool, and (iii) randomize individuals to take part in the MAVEN Initiative or serve in a monitoring group. In the first year, two groups of 20 women will respectively take part in either the MAVEN Initiative or serve in a monitoring group (i.e., a total of 40 women scientists will be involved).

**Figure 2.**
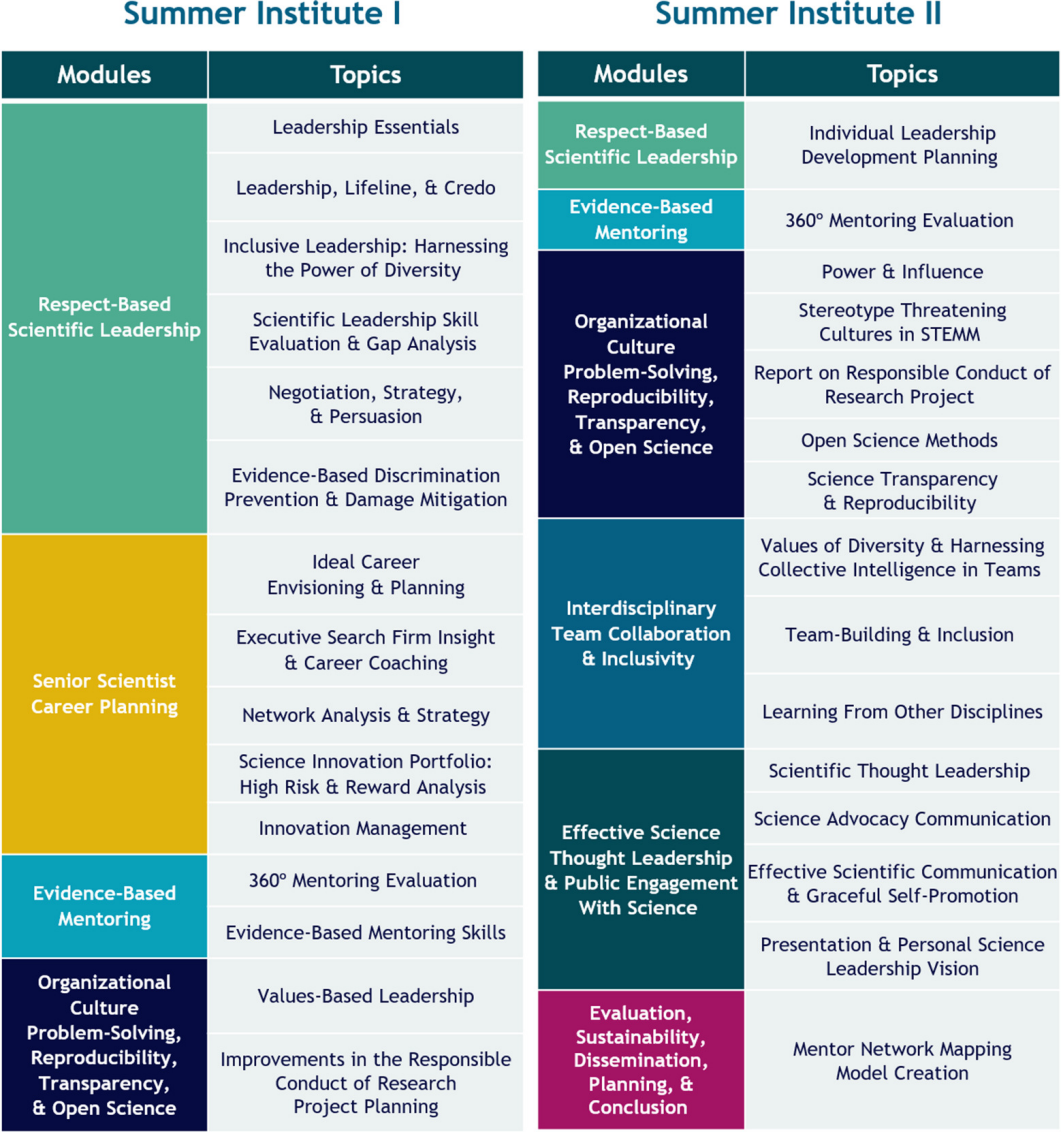
MAVEN summer institutes. The MAVEN Initiative will run two summer institutes. The institutes will have modules on respect-based scientific leadership; senior scientist career planning; evidence-based mentoring; organizational culture problem-solving, reproducibility, transparency, and open science; interdisciplinary team collaboration and inclusivity; effective science thought leadership and public engagement with science; and evaluation, sustainability, dissemination, planning, and conclusion. The topics to be covered within each module are shown in the figure.

Those taking part in MAVEN will be asked to attend two virtual summer institutes that cover a wide range of subjects including respect-based scientific leadership and evidence-based mentoring (Figure 2). Didactic sessions and group exercises will be taught by faculty who specialize in executive leadership, organizational psychology, education, and many other fields, and these sessions and exercises will be complemented by a range of individual and team-based interactive activities. The program is to feature 10 months of virtual mentorship and networking that will include regular meetings with individual mentors to discuss career goal progress, and mentoring pods in which to share career and leadership problems and discuss novel solutions. These pods will be facilitated by professional mentors who will model best practices and ensure that the principles of respect-based leadership are enacted. At their home institutions, participants will be required to complete projects designed to apply what they have been taught about organizational leadership and change.

To determine if the program is functioning as intended, we will use four measures to compare the MAVEN cohort with the monitoring group: (i) career satisfaction (the primary outcome) as assessed by the C-Change faculty survey (Pololi et al., 2015); (ii) peak academic productivity as assessed with the Major Educational Score Index (which has been specifically developed for MAVEN – see below for details); (iii) size of scientific networks as assessed by co-authorship network analysis and n-gram; and (iv) leadership ascension (as assessed by natural language processing analyses of a priori–coded job titles, extracted annually from resumes). The Major Educational Score Index, which will be used to assess productivity, is calculated by summing four normalized variables: h-index (Hirsch, 2005), annual relative citation ratio (Hutchins et al., 2016), annual number of publications in journals with an impact factor above 5, and annual federal grant income. The aim is to increase career satisfaction scores by 10% and network size by 5% compared to the monitoring group after 20 months.

The MAVEN Initiative, like any program of this nature, also carries a number of risks. First, the scientists selected may not advance according to the four measures we are using, which would suggest that the program is deficient in some way. Second, the Initiative might be undersubscribed. However, the 40 available slots for our first cohort (20 to take part in the Initiative and 20 for the monitoring group) were filled within only a few days – this bodes well for the future. Third, the Initiative requires local institutional support; toward this end, we send formal letters to the deans/presidents of invited participants and will provide them with annual updates about the successes of MAVEN. Finally, we have an advisory committee that can guide us in eluding these risks and any other problems or issues that emerge over the five years of the Initiative.

Increasing diversity in the leadership of the scientific workforce is a pressing national and international priority, but it may only be achieved when women and scientists from underrepresented groups can arrive at productive and rewarding leadership positions. It will require the creation of a diverse pipeline of talents, led by role models who demonstrate essential leadership values and ethics, to respect all who contribute to science.

## Data Availability

No new data were generated in this study.
